# Sociodemographic and socioeconomic differences in sleep duration and insomnia-related symptoms in Finnish adults

**DOI:** 10.1186/1471-2458-12-565

**Published:** 2012-07-28

**Authors:** Tea Lallukka, Laura Sares-Jäske, Erkki Kronholm, Katri Sääksjärvi, Annamari Lundqvist, Timo Partonen, Ossi Rahkonen, Paul Knekt

**Affiliations:** 1 Hjelt Institute, Department of Public Health, University of Helsinki, Helsinki, Finland; 2 Centre for Research on Ageing and Gender (CRAG), Department of Sociology, University of Surrey, Guildford, UK; 3 National Institute for Health and Welfare, Helsinki and Turku, Finland

**Keywords:** Marital status, Parental status, Education, Employment status, Household income, Residential area, Insomnia-related symptoms, Sleep duration, Life course, Self-perceived health

## Abstract

**Background:**

Poor sleep tends to be patterned by sociodemographic and socioeconomic factors. The aim of this study was to examine the associations of sociodemographic and socioeconomic factors with sleep duration and insomnia-related symptoms across life course.

**Methods:**

We used cross-sectional Health 2000 Survey (2000–2001) among a total of 5,578 adult Finns, aged 30–79 years, representative of adult Finnish population. Data about sociodemographic and socioeconomic circumstances, insomnia-related symptoms over the previous month as well as average sleep duration were collected by questionnaires. Multinomial logistic regression models were adjusted first for gender and age, second for sociodemographic factors, third additionally for socioeconomic factors, and fourth for all covariates and self-perceived health simultaneously.

**Results:**

On average 70% of Finnish adults slept 7–8 hours a day. Frequent insomnia-related symptoms were more prevalent among women (14%) than men (10%). Not being married, not having children, having low education, low income, being unemployed, and being a disability retiree were associated with frequent insomnia-related symptoms. Similar factors were associated with short and long sleep duration. However, childhood socioeconomic position was mostly unrelated to sleep in adulthood except parental education had some associations with short sleep duration.

**Conclusions:**

Disadvantaged socioeconomic position in adulthood, in particular income and employment status, is associated with poorer sleep. When promoting optimal sleep duration and better sleep quality, families with low incomes, unemployed people, and disability retirees should be targeted.

## Background

Both sleep duration and quality tend to vary in populations as a function of sociodemographic and socioeconomic factors [1-7]. In order to shed light on the social determinants of sleep, a broad approach simultaneously covering a variety of indicators of sociodemographic and socioeconomic factors across life course is needed. First, life course perspective is important because adverse childhood conditions may predict sleep problems in adulthood [7,8]. Second, married people tend to sleep better [1-3,9] and slightly longer [10,11] than those living alone. However, qualitative research has reported that snoring and other sleep disruptions caused by a partner could be related to poorer sleep [12]. Third, especially young [13] but also teenage children [14] can disrupt their parent’s sleep. Fourth, sleep may vary according to residential area [11]. For example, people living in urban areas could be more likely to have poor sleep. Fifth, sleep duration and quality tend to vary by education, occupational class, income, and employment status [1-3,9,15]. Thus, those at the lower end of the social structure tend to have poorer sleep.

Although these many studies have suggested that sleep deprivation is patterned by social factors, the associations between sociodemographic and socioeconomic factors and sleep, described above are, however, inconsistent, i.e., not all have found differences in insomnia by conventional indicators of socioeconomic position, for example [7]. Moreover, nationally representative studies focusing on a broad variety of sociodemographic and socioeconomic factors as determinants of both sleep duration and insomnia-related symptoms over life course are sparse. Further evidence is needed because insomnia is the most prevalent sleep problem, and it is associated with key public health problems [16,17], work disability [18,19], health care use [20,21], and mortality [22]. In Finland, insomnia is even more prevalent than in many other European countries [23] highlighting the importance to shed light on social patterning of sleep in the Finnish general population. In addition to a decline in well-being and health, the indirect and direct costs and societal burden of insomnia are, therefore, substantial [24-27].

Evidence concerning sleep duration, morbidity, and mortality is even more extensive. The first study showing the U-shaped association between sleep duration and increased mortality was published almost 50 years ago [28], and the study has been followed by dozens of others [29-33].

Our study is an extension of a previous study that focused on sleep duration in the general Finnish population [34]. The study did not cover childhood socioeconomic position to take into account life course perspective. Neither was income included. Furthermore, in this study, we took into account health status and focused on the extreme ends of sleep duration distribution where health and mortality risks have been shown to be the highest [35]. We also included insomnia-related symptoms which are associated with morbidity and mortality over and above the contribution of sleep duration [22,36-38]. New evidence regarding the associations among sleep and sociodemographic and socioeconomic circumstances is vital for the identification of key risk groups for sub-optimal sleep duration and poor sleep quality. Such evidence can be used to prevent insomnia-related symptoms and promote better public health.

The aim in this study was to simultaneously examine several sociodemographic and socioeconomic differences in sleep duration and insomnia-related symptoms using data representative of the general adult Finnish population.

## Methods

### Data

Data for this study were collected from a nationally representative cross-sectional health survey (Health 2000 Survey), which was conducted between 2000 and 2001, mainly during autumn and winter, among adult Finns aged 30 or over [39-42]. The sample (n = 8028) was formed using a 2-stage stratified cluster sampling design, which was planned by Statistics Finland. After excluding those who died before health examinations (n = 49), the final size of the target population was 7979. All 30–79-year-olds who had participated in home-interview (response rate 89%) and health examination (85%), and had returned questionnaires (79%) which included questions about sleep duration, insomnia-related symptoms, and covariates, were included in this study (n = 5578, 70% of the final target population) [42]. Four questionnaires were collected: one questionnaire was mailed to the participants before their health interviews, another questionnaire was filled in during the health examination, and two questionnaires were given to participants after the health examination and were returned by mail. More details about the data collection and methods can be found elsewhere [39-41], as well as at the project website: http://www.terveys2000.fi/indexe.html. A flow chart of the Health 2000 study questionnaire data collection and health examinations has been published as part of the Methodology report [42]. The Ethics Committee for Epidemiology and Public Health of the hospital district of Helsinki and Uusimaa in Finland approved the Health 2000 Survey.

### Sleep duration and insomnia-related questions

Average sleep duration was based on responses to a question: “How many hours do you sleep in 24 hours”. No time frame was specified. Based on earlier literature [29,35,39] about the U-shaped association between sleep duration and health-related outcomes the responses were classified into seven categories: <5 hours, 5 hours, 6 hours, 7 hours, 8 hours, 9 hours, and ≥10 hours, and 7 hour sleepers were used as a reference group in the analyses.

Insomnia-related symptoms were assessed by a single question which originated from the Symptom Checklist-90 (SCL-90) questionnaire [43]. In the questionnaire, the respondents are asked if they have had general symptoms or problems during the preceding 30 days. Insomnia and disturbed sleep were asked as part of the symptoms check-list. To assess insomnia-related symptoms, a following question was asked: ‘During the past month (30 days), how often have you been bothered by disturbed sleep or insomnia?’ Five response alternatives were given: not at all; a little, to some extent; quite often and very often. The response alternatives were reclassified into three groups: no (answering modes ‘not at all’ and ‘a little’), occasional (answering mode ‘to some extent’), and frequent (answering modes ‘quite often’ and ‘very often’). Three categories were formed to be able to better show a gradient in the associations between sociodemographic and socioeconomic factors and insomnia symptoms, and because even milder and occasional symptoms are related to poorer health and work ability [18,19]. In addition to insomnia-related symptoms, frequency of hypnotic use was requested. The responses were used in sensitivity analyses.

### Sociodemographic factors

Gender was a covariate in all the analyses, with men as a reference group. Age was categorized into five 10-year age groups: 30–39 years (reference), 40–49 years, 50–59 years, 60–69 years, and 70–79 years. Marital status was classified into four groups: married or cohabiting (reference), single, divorced or separated, and widowed. Sensitivity analyses were conducted separating those married and cohabiting (data not shown). However, the results for the married and cohabiting followed similar patterns and thus we preferred to combine the groups and compare those living with a partner to those living alone. Respondents were also asked whether they had children living in the same household and the ages of the children. Respondents without children were compared to those who lived with children aged ≤7 years, those living with children aged 7–17 years, or living with both small and older children. Residential area was classified as urban town (reference), densely populated municipality, or rural municipality. The type of municipality is based on its population density and is classified by Statistics Finland.

### Socioeconomic circumstances

Childhood socioeconomic position (low, intermediate, and high) was determined by the highest educational level of either parent and was self-reported by the participants. Educational class was based on Finnish educational system at a time. There were six response alternatives. The first three represented very low or low level of education (elementary school). The intermediate level referred to middle school education without any high school training. The highest educational level referred to parts of or completed high school level or university degree. Own education was determined according to years of completed education: very low (≤7 years), low (7–9 years), medium (10–12 years), and high (≥12 years) education. Due to notable changes in the educational system, educational levels between respondents and their parents are not comparable as such but indicators of childhood and own education reflect the hierarchy in the society. Household income was derived using the tax administration register and scaled using the number of consumption units in a household. Employment status was classified as working (full-time or part-time), unemployed or laid off, retired due to old age, retired due to disability, retired due to other reason, and others (students, housewives, conscripts etc.)

### Health status

Health status was defined by self-perceived health which has shown to have good reliability across population subgroups [44] and to be connected to both sleep duration [45] and insomnia-related symptoms [3]. Additionally, self-perceived health is a predictor of morbidity and mortality [46] as well as disability retirement [47]. Respondents were asked to assess their current health status with five response alternatives ranging from poor to good self-perceived health.

### Statistical analyses

First, we computed gender and age-adjusted prevalence (with 95% Confidence Intervals, 95% CI) for sleep duration and insomnia-related symptoms by each sociodemographic and socioeconomic factor (Tables 1 and 2). Sampling design was taken into account to produce an estimate for the general population.

**Table 1:** Sleep duration: distributions of variables, prevalence table

		**Sleep duration**^**1**^						
		**<5 h**	**5 h**	**6 h**	**7 h**	**8 h**	**9 h**	**≥10 h**
	**N**^**2**^	**% (95% CI)**	**% (95% CI)**	**% (95% CI)**	**% (95% CI)**	**% (95% CI)**	**% (95% CI)**	**% (95% CI)**
**Gender**^**3**^	5578							
Male	2542	4.92 (4.16, 5.80)	3.03 (2.40, 3.82)	12.5 (11.2, 13.8)	37.9 (36.0, 39.8)	33.6 (31.9, 35.4)	5.55 (4.66, 6.52)	2.52 (1.98, 3.20)
Female	3036	4.26 (3.56, 5.09)	2.63 (2.15, 3.22)	9.77 (8.67, 11.0)	32.6 (31.0, 34.3)	38.1 (36.4, 39.8)	9.73 (8.76, 10.8)	2.93 (2.41, 3.57)
**Age**^**4**^	5578							
30–39	1343	2.10 (1.45, 3.04)	1.28 (0.81, 2.01)	9.07 (7.72, 10.6)	34.1 (31.5, 36.9)	40.5 (37.8, 43.3)	11.3 (9.75, 13.0)	1.59 (1.05, 2.42)
40–49	1443	2.87 (2.01, 3.92)	2.23 (1.66, 2.98)	10.0 (8.64, 11.5)	37.7 (35.1, 40.4)	38.9 (36.3, 41.5)	6.17 (5.12, 7.41)	2.17 (1.54, 3.05)
50–59	1311	4.69 (3.61, 6.06)	3.37 (2.49, 4.56)	12.6 (10.7, 14.6)	38.0 (35.6, 40.5)	34.2 (31.6, 36.8)	4.90 (3.87, 6.18)	2.29 (1.58, 3.30)
60–69	910	5.84 (4.50, 7.54)	3.41 (2.37, 4.89)	10.4 (8.56, 12.6)	33.5 (30.6, 36.6)	33.6 (30.6, 36.6)	9.10 (7.41, 11.1)	4.12 (2.97, 5.69)
70–79	571	11.8 (9.54, 14.6)	5.41 (3.85, 7.56)	15.4 (12.4, 18.9)	26.8 (23.6, 30.2)	26.9 (23.2, 30.9)	8.18 (5.86, 11.3)	5.47 (3.94, 7.57)
**Parental education**	5445							
High	338	3.11 (1.55, 6.12)	0.77 (0.19, 2.98)	11.6 (8.60, 15.4)	35.9 (31.0, 41.1)	37.7 (32.5, 43.2)	9.48 (6.86, 13.0)	1.50 (0.59, 3.76)
Intermediate	432	3.10 (1.65, 5.77)	3.87 (2.25, 6.57)	9.77 (7.12, 13.2)	34.8 (30.8, 39.1)	36.7 (32.2, 41.4)	7.78 (5.67, 10.6)	3.96 (2.34, 6.60)
Low	4581	4.56 (3.97, 5.22)	2.84 (2.38, 3.39)	11.2 (10.3, 12.1)	35.3 (33.9, 36.7)	35.8 (34.4, 37.2)	7.64 (6.91, 8.45)	2.72 (2.27, 3.26)
Do not know	94	6.29 (2.99, 12.7)	3.76 (1.46, 9.34)	6.34 (3.02, 12.9)	37.8 (28.7, 47.8)	36.8 (28.4, 46.0)	8.12 (3.92, 16.1)	0.89 (0.12, 6.27)
**Marital status**	5578							
Married/living together	4060	4.05 (3.40, 4.80)	2.49 (2.03, 3.05)	9.82 (8.87, 10.9)	35.9 (34.3, 37.6)	37.5 (36.2, 38.9)	7.72 (6.92, 8.61)	2.44 (1.98, 3.00)
Single	621	6.04 (4.32, 8.39)	2.42 (1.43, 4.08)	13.1 (10.5, 16.1)	31.6 (28.1, 35.3)	32.8 (29.1, 36.7)	9.18 (7.20, 11.6)	4.90 (3.37, 7.07)
Divorced/separated	553	6.35 (4.55, 8.80)	4.08 (2.78, 5.95)	15.5 (12.8, 18.6)	33.1 (29.7, 36.6)	29.8 (26.1, 33.8)	7.51 (5.70, 9.83)	3.64 (2.38, 5.53)
Widowed	344	4.78 (3.23, 7.01)	4.44 (2.76, 7.06)	14.4 (11.0, 18.5)	35.4 (29.9, 41.2)	33.5 (28.5, 38.9)	5.45 (3.39, 8.65)	2.10 (1.18, 3.68)
**Number of children**	5568							
No under 18 years old children	3633	4.77 (4.16, 5.46)	2.94 (2.42, 3.56)	11.5 (10.3, 12.7)	34.6 (32.8, 36.3)	35.4 (33.7, 37.2)	7.80 (6.87, 8.84)	3.03 (2.52, 3.63)
Under 7-year-olds	414	3.85 (1.79, 8.07)	0.44 (0.06, 3.32)	9.78 (6.93, 13.6)	35.5 (31.0, 40.2)	39.0 (34.0, 44.2)	10.1 (7.33, 13.7)	1.41 (0.52, 3.71)
7–17-year-olds	1125	3.93 (2.73, 5.62)	2.57 (1.60, 4.11)	10.7 (8.98, 12.7)	37.6 (34.8, 40.5)	36.8 (34.1, 39.5)	6.42 (5.14, 7.99)	1.96 (1.21, 3.17)
Both	396	3.52 (1.73, 7.04)	4.17 (2.16, 7.90)	8.99 (6.15, 13.0)	36.1 (31.4, 41.1)	37.4 (32.7, 42.4)	8.40 (5.96, 11.7)	1.38 (0.50, 3.76)
**Own education (years)**	5560							
high (> 12)	2045	2.71 (1.96, 3.73)	1.60 (1.06, 2.40)	10.1 (8.82, 11.6)	37.0 (34.8, 39.2)	38.6 (36.4, 40.9)	8.11 (7.05, 9.32)	1.87 (1.32, 2.66)
medium (10–12)	1551	4.90 (3.85, 6.21)	3.70 (2.78, 4.89)	10.9 (9.46, 12.6)	36.5 (33.8, 39.4)	34.2 (31.8, 36.8)	6.84 (5.76, 8.10)	2.85 (2.05, 3.94)
low (7–9)	1559	5.54 (4.48, 6.83)	3.22 (2.51, 4.12)	11.7 (10.0, 13.7)	33.3 (30.4, 36.4)	35.1 (32.6, 37.7)	8.00 (6.61, 9.64)	3.08 (2.37, 4.01)
very low (< 7)	405	5.56 (3.92, 7.83)	2.96 (1.80, 4.83)	13.3 (10.1, 17.3)	30.4 (25.5, 35.8)	34.0 (29.1, 39.2)	9.75 (6.33, 14.7)	4.10 (2.68, 6.21)
**Household income level**	5453							
Highest income quartile	1491	3.80 (2.91, 4.95)	2.32 (1.60, 3.35)	10.4 (8.95, 11.9)	40.4 (38.0, 42.9)	35.9 (33.4, 38.5)	5.89 (4.84, 7.20)	1.28 (0.82, 2.00)
3^rd^	1528	4.25 (3.30, 5.45)	2.52 (1.72, 3.67)	10.6 (9.07, 12.4)	35.5 (32.7, 38.4)	37.4 (35.0, 39,9)	8.06 (6.82, 9.50)	1.59 (1.04, 2.40)
2^nd^	1314	5.13 (4.09, 6.42)	2.97 (2.16, 4.05)	11.2 (9.50, 13.0)	34.0 (31.5, 36.5)	35.6 (33.1, 38.1)	7.85 (6.43, 9.54)	3.38 (2.60, 4.39)
Lowest income quartile	1120	5.15 (4.10, 6.45)	3.86 (2.98, 4.99)	11.9 (10.1, 13.8)	29.8 (27.2, 32.5)	34.7 (31.7, 37.7)	10.0 (8.28, 12.0)	4.68 (3.62, 6.03)
**Employment status**	5578							
Working	3363	4.44 (3.39, 5.81)	2.67 (1.93, 3.70)	11.7 (10.4, 13.1)	39.4 (37.3, 41.6)	35.7 (33.9, 37.5)	5.27 (4.55, 6.09)	0.83 (0.58, 1.17)
Unemployed	420	6.53 (4.21, 9.97)	3.80 (2.21, 6.43)	10.3 (7.48, 14.0)	31.8 (27.5, 36.4)	33.6 (29.2, 38.2)	9.20 (7.03, 12.0)	4.81 (3.16, 7.24)
Disability pension	427	5.74 (4.01, 8.15)	4.33 (2.78, 6.70)	11.1 (8.51, 14.4)	24.9 (20.9, 29.3)	31.2 (27.0, 35.7)	13.7 (9.65, 19.1)	8.97 (6.10, 13.0)
Old age retirement	1022	3.86 (2.94, 5.06)	2.45 (1.65, 3.63)	9.35 (7.45, 11.7)	25.5 (21.9, 29.5)	35.2 (30.7, 40.0)	18.1 (13.2, 24.4)	5.44 (3.39, 8.61)
Other pension	135	5.06 (2.78, 9.03)	1.08 (0.28, 4.08)	6.90 (3.79, 12.2)	34.1 (26.1, 43.1)	37.0 (28.2, 46.7)	12.1 (6.92, 20.3)	3.76 (1.54, 8.90)
Other	211	3.87 (1.49, 9.65)	2.41 (0.72, 7.72)	9.15 (5.74, 14.3)	27.0 (20.8, 34.2)	40.7 (33.4, 48.3)	11.8 (8.71, 15.8)	5.12 (2.82, 9.12)
**Residential area**	5578							
Urban town	3421	4.23 (3.60, 4.96)	2.63 (2.14, 3.24)	11.5 (10.5, 12.6)	36.2 (34.5, 37.9)	35.5 (34.0, 37.0)	7.66 (6.81, 8.59)	2.33 (1.91, 2.84)
Densely populated municipality	805	5.14 (4.06, 6.48)	2.95 (2.15, 4.01)	9.50 (7.17, 12.5)	34.0 (31.1, 37.0)	37.7 (34.5, 40.8)	7.55 (5.89, 9.63)	3.20 (2.37, 4.31)
Rural municipality	1352	5.01 (3.92, 6.38)	3.22 (2.29, 4.52)	10.8 (9.13, 12.6)	33.1 (30.9, 35.4)	35.7 (33.5, 38.0)	8.70 (7.39, 10.2)	3.47 (2.50, 4.79)
**Self-perceived health**	5568							
Good	1899	3.02 (2.21, 4.10)	1.92 (1.34, 2.75)	9.90 (8.58, 11.4)	37.9 (35.7, 40.2)	38.2 (36.1, 40.4)	7.77 (6.60, 9.04)	1.28 (0.85, 1.92)
Quite good	1709	4.21 (3.37, 5.25)	1.73 (1.19, 2.52)	10.9 (9.38, 12.5)	37.0 (34.8, 39.3)	36.4 (34.4, 38.6)	7.56 (6.52, 8.74)	2.20 (1.64, 2.93)
Mediocre	1425	4.78 (3.91, 5.85)	3.40 (2.63, 4.38)	12.5 (10.8, 14.3)	32.5 (30.2, 35.0)	35.3 (32.9, 37.8)	7.75 (6.38, 9.38)	3.74 (2.98, 4.68)
Quite poor	401	5.61 (3.85, 8.09)	5.23 (3.41, 7.95)	13.3 (10.2, 17.2)	31.5 (27.3, 36.1)	31.1 (26.5, 36.0)	7.71 (5.37, 10.9)	5.51 (3.58, 8.37)
Poor	134	16.6 (10.6, 24.9)	10.6 (6.61, 16.5)	9.58 (5.68, 15.7)	18.8 (12.9, 26.6)	23.7 (16.7, 32.5)	12.9 (8.19, 19.8)	7.83 (4.40, 13.5)
*Total (N)*	*5578*	*251*	*156*	*605*	*1942*	*2018*	*451*	*155*

^1^ Adjusted for age and gender, ^2^ Depending on variable missing information n = 0–133, ^3^ Adjusted for age, ^4^ Adjusted for gender

**Table 2:** Insomnia-related symptoms: distributions of variables, prevalence table

		**Insomnia-related symptoms**^**1**^		
		**No**	**Occasional**	**Frequent**
	**N**^**2**^	**% (95% CI)**	**% (95% CI)**	**% (95% CI)**
**Gender**^3^	5578			
Male	2542	69.3 (67.6, 71.1)	20.6 (19.1, 22.2)	10.0 (9.00, 11.2)
Female	3036	63.0 (61.2, 64.7)	22.9 (21.4, 24.5)	14.1 (13.0, 15.3)
**Age**^4^	5578			
30–39	1343	76.7 (74.3, 79.0)	16.8 (14.9, 18.9)	6.51 (5.29, 7.98)
40–49	1443	69.4 (66.9, 71.8)	20.7 (18.7, 22.9)	9.85 (8.49, 11.4)
50–59	1311	61.9 (59.0, 64.7)	22.8 (20.4, 25.4)	15.3 (13.4, 17.4)
60–69	910	59.8 (56.8, 62.8)	24.3 (21.7, 27.2)	15.8 (13.5, 18.5)
70–79	571	53.1 (49.5, 56.7)	29.3 (26.1, 32.7)	17.6 (14.8, 20.9)
**Parental education**	5445			
High	338	68.0 (62.8, 72.7)	20.4 (16.3, 25.3)	11.6 (8.51, 15.6)
Intermediate	432	68.6 (64.2, 72.7)	18.6 (15.3, 22.5)	12.8 (9.80, 16.4)
Low	4581	65.7 (64.3, 67.2)	22.3 (21.1, 23.6)	11.9 (11.1, 12.9)
Do not know	94	71.4 (62.3, 79.0)	11.2 (6.35, 19.1)	17.4 (11.5, 25.4)
**Marital status**	5578			
Married/living together	4060	67.5 (66.1, 68.8)	21.0 (19.8, 22.2)	11.6 (10.7, 12.5)
Single	621	63.9 (59.9, 67.7)	24.4 (21.1, 28.0)	11.7 (9.21, 14.8)
Divorced/separated	553	61.8 (57.5, 65.9)	21.5 (18.3, 25.2)	16.7 (13.5, 20.5)
Widowed	344	59.3 (54.0, 64.5)	27.4 (22.5, 32.9)	13.3 (10.5, 16.7)
**Number of children**	5568			
No under 18 years old children	3633	63.7 (62.0, 65.3)	22.6 (21.2, 24.2)	13.7 (12.6, 14.9)
Under 7-year-olds	414	72.5 (67.5, 77.0)	22.7 (18.4, 27.5)	4.82 (2.91, 7.91)
7–17-year-olds	1125	69.8 (66.9, 72.5)	19.9 (17.6, 22.4)	10.3 (8.64, 12.2)
Both	396	72.9 (67.8, 77.5)	19.0 (15.0, 23.6)	8.10 (5.64, 11.5)
**Own education (years)**	5560			
high (> 12)	2045	68.6 (66.6, 70.6)	21.6 (19.8, 23.6)	9.76 (8.37, 11.4)
medium (10–12)	1551	65.3 (62.5, 68.0)	21.6 (19.4, 24.0)	13.1 (11.3, 15.0)
low (7–9)	1559	63.3 (60.8, 65.7)	22.8 (20.8, 25.0)	13.9 (12.2, 15.7)
very low (< 7)	405	67.4 (62.7, 71.8)	20.5 (17.0, 24.6)	12.1 (9.55, 15.1)
**Household income level**	5453			
Highest income quartile	1491	68.9 (66.4, 71.3)	21.3 (19.2, 23.4)	9.88 (8.43, 11.6)
3^rd^	1528	69.4 (67.1, 71.7)	20.3 (18.3, 22.5)	10.2 (8.69, 12.0)
2^nd^	1314	63.1 (60.5, 65.7)	23.7 (21.5, 25.9)	13.2 (11.7, 15.0)
Lowest income quartile	1120	60.5 (57.8, 63.1)	23.4 (20.8, 26.1)	16.2 (14.2, 18.4)
**Employment status**	5578			
Working	3363	68.9 (66.8, 71.0)	21.6 (19.9, 23.5)	9.46 (8.16, 10.9)
Unemployed	420	53.2 (48.6, 57.6)	24.3 (20.6, 28.5)	22.5 (18.6, 27.0)
Disability pension	427	54.0 (48.8, 59.2)	22.2 (18.5, 26.6)	23.7 (19.7, 28.3)
Old age retirement	1022	68.4 (64.3, 72.3)	20.8 (17.6, 24.3)	10.8 (8.67, 13.4)
Other pension	135	66.7 (59.2, 73.4)	20.6 (15.3, 27.0)	12.8 (8.40, 18.9)
Other	211	58.7 (50.9, 66.0)	27.9 (21.6, 35.2)	13.4 (8.78, 19.9)
**Residential area**	5578			
Urban town	3421	65.7 (64.0, 67.3)	22.0 (20.5, 23.5)	12.4 (11.2, 13.5)
Densely populated municipality	805	66.7 (64.0, 69.2)	21.8 (19.5, 24.2)	11.6 (9.67, 13.8)
Rural municipality	1352	66.3 (63.6, 68.8)	21.4 (19.3, 23.8)	12.3 (11.0, 13.7)
**Self-perceived health**	5568			
Good	1899	79.9 (78.0, 81.6)	15.4 (13.9, 17.1)	4.71 (3.89, 5.70)
Quite good	1709	68.0 (65.8, 70.0)	22.8 (20.8, 25.0)	9.19 (7.92, 10.6)
Mediocre	1425	56.9 (54.1, 59.7)	27.3 (25.0, 29.8)	15.7 (13.9, 17.8)
Quite poor	401	40.3 (35.6, 45.2)	25.7 (21.4, 30.5)	34.0 (29.0, 39.4)
Poor	134	31.1 (23.4, 40.1)	23.0 (16.2, 31.6)	45.9 (36.7, 55.4)
*Total (N)*	*5578*	*3690*	*1212*	*676*

^1^ Adjusted for age and gender, ^2^ Depending on variable missing information n = 0–133, ^3^ Adjusted for age, ^4^ Adjusted for gender.

Second, we conducted multinomial logistic regression analyses (Odds Ratios*, OR*, and their 95% *CI*) to examine the associations between sociodemographic and socioeconomic factors and sleep duration, and insomnia-related symptoms (Tables 3, 4, 5, 6). We fitted proportional odds models using a multilog procedure with a cumulative logit link function. For each indicator, the reference (*OR* 1.00) was the highest/most advantaged sociodemographic or socioeconomic group.

**Table 3:** Sleep duration: associations with sociodemographic factors

	**Model 1 Adjusted for age and gender**		**Model 2 Adjusted for age, gender, marital status, children and area**		**Model 3 Adjusted for age, gender, marital status, children, area, education, parental education, employment status and income**		**Model 4 Adjusted for age, gender, marital status, children, area, education, parental education, employment status, income and self-perceived health**	
	**OR (95% CI)**	**P-value**	**OR (95% CI)**	**P-value**	**OR (95% CI)**	**P-value**	**OR (95% CI)**	**P-value**
**MARITAL STATUS**								
**< 5 h vs. 7 h**								
Married/living together	1		1		1		1	
Single	1.74 (1.15, 2.64)	0.009	1.68 (1.09, 2.58)	0.02	1.43 (0.89, 2.31)	0.14	1.45 (0.90, 2.36)	0.13
Divorced/separated	1.76 (1.13, 2.72)	0.01	1.79 (1.16, 2.75)	0.009	1.82 (1.16, 2.86)	0.009	1.70 (1.06, 2.71)	0.03
Widowed	1.22 (0.73, 2.03)	0.45	1.20 (0.72, 2.01)	0.49	1.23 (0.73, 2.08)	0.43	1.28 (0.76, 2.18)	0.35
**5 h vs. 7 h**								
Married/living together	1		1		1		1	
Single	1.13 (0.62, 2.03)	0.69	1.05 (0.56, 1.98)	0.88	0.91 (0.48, 1.72)	0.76	0.92 (0.48, 1.76)	0.80
Divorced/separated	1.82 (1.11, 2.97)	0.02	1.80 (1.11, 2.92)	0.02	1.48 (0.89, 2.47)	0.13	1.28 (0.76, 2.13)	0.35
Widowed	1.83 (1.07, 3.15)	0.03	1.82 (1.06, 3.14)	0.03	1.79 (1.02, 3.14)	0.04	1.91 (1.09, 3.36)	0.02
**6 h vs. 7 h**								
Married/living together	1		1		1		1	
Single	1.52 (1.13, 2.05)	0.006	1.48 (1.06, 2.08)	0.02	1.50 (1.07, 2.09)	0.02	1.50 (1.07, 2.10)	0.02
Divorced/separated	1.73 (1.34, 2.23)	<0.0001	1.70 (1.30, 2.21)	0.0001	1.66 (1.26, 2.17)	0.0003	1.66 (1.26, 2.18)	0.0003
Widowed	1.49 (1.03, 2.16)	0.03	1.49 (1.03, 2.15)	0.04	1.37 (0.93, 2.02)	0.11	1.39 (0.95, 2.05)	0.09
**8 h vs. 7 h**								
Married/living together	1		1		1		1	
Single	0.99 (0.80, 1.23)	0.95	0.98 (0.78, 1.24)	0.88	0.91 (0.71, 1.15)	0.42	0.91 (0.71, 1.15)	0.41
Divorced/separated	0.86 (0.70. 1.06)	0.16	0.86 (0.70, 1.07)	0.18	0.86 (0.68, 1.09)	0.21	0.87 (0.69, 1.10)	0.24
Widowed	0.90 (0.67, 1.22)	0.50	0.89 (0.66, 1.20)	0.44	0.87 (0.63, 1.20)	0.39	0.89 (0.64, 1.22)	0.46
**9 h vs. 7 h**								
Married/living together	1		1		1		1	
Single	1.35 (0.99, 1.84)	0.06	1.36 (0.98, 1.88)	0.07	1.09 (0.76, 1.56)	0.63	1.09 (0.76, 1.56)	0.65
Divorced/separated	1.05 (0.74, 1.49)	0.78	1.09 (0.76, 1.55)	0.64	1.04 (0.72, 1.52)	0.83	1.04 (0.71, 1.51)	0.85
Widowed	0.71 (0.41, 1.23)	0.23	0.64 (0.37, 1.11)	0.11	0.57 (0.32, 1.03)	0.06	0.59 (0.33, 1.05)	0.07
**≥ 10 h vs. 7 h**								
Married/living together	1		1		1		1	
Single	2.33 (1.51, 3.58)	0.0001	2.04 (1.30, 3.22)	0.002	1.63 (1.01, 2.66)	0.05	1.66 (1.03, 2.70)	0.04
Divorced/separated	1.66 (0.99, 2.77)	0.05	1.62 (0.96, 2.73)	0.07	1.51 (0.86, 2.63)	0.15	1.45 (0.83, 2.54)	0.19
Widowed	0.88 (0.45, 1.74)	0.72	0.82 (0.41, 1.64)	0.57	0.85 (0.42, 1.73)	0.66	0.87 (0.42, 1.77)	0.69
**Global P-value**	<0.0001		<0.0001		<0.0001		0.0001	
**n**	5578		5568		5307		5300	
**NUMBER OF CHILDREN**								
**< 5 h vs. 7 h**								
No under 18 years old children	1		1		1		1	
Under 7-year-olds	0.77 (0.34, 1.74)	0.53	0.94 (0.41, 2.14)	0.89	0.98 (0.40, 2.38)	0.96	1.04 (0.43, 2.48)	0.93
7–17-year-olds	0.75 (0.49, 1.16)	0.19	0.84 (0.54, 1.29)	0.42	0.83 (0.51, 1.33)	0.44	0.83 (0.52, 1.34)	0.45
Both	0.70 (0.32, 1.52)	0.37	0.83 (0.38, 1.81)	0.64	0.83 (0.36, 1.91)	0.67	0.91 (0.40, 2.08)	0.82
**5 h vs. 7 h**								
No under 18 years old children	1		1		1		1	
Under 7-year-olds	0.14 (0.02, 1.15)	0.07	0.15 (0.02, 1.23)	0.08	0.13 (0.02, 1.04)	0.05	0.14 (0.02, 1.10)	0.06
7–17-year-olds	0.80 (0.46, 1.39)	0.43	0.80 (0.45, 1.43)	0.46	0.70 (0.39, 1.26)	0.24	0.71 (0.40, 1.29)	0.26
Both	1.35 (0.63, 2.92)	0.44	1.38 (0.62, 3.07)	0.43	1.12 (0.51, 2.45)	0.78	1.25 (0.56, 2.76)	0.58
**6 h vs. 7 h**								
No under 18 years old children	1		1		1		1	
Under 7-year-olds	0.83 (0.55, 1.25)	0.37	0.99 (0.63, 1.53)	0.95	0.92 (0.57, 1.49)	0.73	0.94 (0.58, 1.53)	0.82
7–17-year-olds	0.86 (0.66, 1.11)	0.24	0.96 (0.72, 1.28)	0.79	0.86 (0.62, 1.18)	0.35	0.86 (0.62, 1.19)	0.37
Both	0.75 (0.47, 1.18)	0.22	0.88 (0.54, 1.43)	0.60	0.79 (0.47, 1.33)	0.37	0.82 (0.48, 1.38)	0.44
**8 h vs. 7 h**								
No under 18 years old children	1		1		1		1	
Under 7-year-olds	1.07 (0.82, 1.40)	0.60	1.06 (0.80, 1.40)	0.70	0.87 (0.65, 1.17)	0.36	0.88 (0.66, 1.18)	0.39
7–17-year-olds	0.95 (0.81, 1.12)	0.56	0.94 (0.79, 1.11)	0.44	0.87 (0.73, 1.05)	0.16	0.88 (0.73, 1.06)	0.16
Both	1.01 (0.78, 1.30)	0.93	0.99 (0.76, 1.30)	0.95	0.83 (0.63, 1.10)	0.20	0.84 (0.63, 1.12)	0.23
**9 h vs. 7 h**								
No under 18 years old children	1		1		1		1	
Under 7-year-olds	1.26 (0.84, 1.91)	0.26	1.44 (0.93, 2.25)	0.10	0.88 (0.55, 1.42)	0.60	0.89 (0.56, 1.44)	0.64
7–17-year-olds	0.76 (0.55, 1.04)	0.08	0.82 (0.59, 1.14)	0.23	0.80 (0.56, 1.15)	0.23	0.81 (0.56, 1.15)	0.24
Both	1.03 (0.66, 1.60)	0.89	1.15 (0.73, 1.80)	0.54	0.84 (0.51, 1.38)	0.49	0.86 (0.53, 1.41)	0.55
**≥ 10 h vs. 7 h**								
No under 18 years old children	1		1		1		1	
Under 7–year-olds	0.45 (0.16, 1.27)	0.13	0.59 (0.20, 1.68)	0.32	0.23 (0.06, 0.80)	0.02	0.25 (0.07, 0.88)	0.03
7–17-year-olds	0.59 (0.35, 0.99)	0.05	0.68 (0.40, 1.15)	0.15	0.75 (0.43, 1.33)	0.33	0.76 (0.44, 1.32)	0.33
Both	0.44 (0.15, 1.29)	0.13	0.54 (0.18, 1.65)	0.28	0.34 (0.11, 1.03)	0.06	0.39 (0.13, 1.19)	0.10
**Global P-value**	0.09		0.45		0.52		0.59	
**n**	5568		5568		5307		5300	
**RESIDENTIAL AREA**								
**< 5 h vs. 7 h**								
Urban town	1		1		1		1	
Densely populated municipality	1.30 (0.94, 1.79)	0.11	1.35 (0.98, 1.87)	0.07	1.32 (0.94, 1.85)	0.11	1.37 (0.96, 1.95)	0.08
Rural municipality	1.30 (0.94, 1.80)	0.11	1.34 (0.96, 1.86)	0.08	1.26 (0.88, 1.81)	0.21	1.28 (0.90, 1.81)	0.17
**5 h vs. 7 h**								
Urban town	1		1		1		1	
Densely populated municipality	1.20 (0.82, 1.75)	0.36	1.28 (0.87, 1.88)	0.21	1.10 (0.76, 1.58)	0.62	1.18 (0.81, 1.71)	0.39
Rural municipality	1.35 (0.86, 2.11)	0.19	1.38 (0.89, 2.15)	0.15	1.29 (0.82, 2.03)	0.27	1.27 (0.80, 2.02)	0.31
**6 h vs. 7 h**								
Urban town	1		1		1		1	
Densely populated municipality	0.88 (0.62, 1.24)	0.47	0.92 (0.65, 1.30)	0.63	0.87 (0.62, 1.23)	0.43	0.87 (0.61, 1.23)	0.43
Rural municipality	1.03 (0.83, 1.28)	0.80	1.06 (0.85, 1.31)	0.60	1.00 (0.79, 1.26)	0.98	0.99 (0.79, 1.25)	0.95
**8 h vs. 7 h**								
Urban town	1		1		1		1	
Densely populated municipality	1.13 (0.96, 1.32)	0.13	1.13 (0.97, 1.33)	0.13	1.09 (0.93, 1.28)	0.28	1.09 (0.93, 1.28)	0.30
Rural municipality	1.10 (0.97, 1.25)	0.14	1.09 (0.96, 1.24)	0.16	1.09 (0.94, 1.25)	0.27	1.08 (0.94, 1.25)	0.28
**9 h vs. 7 h**								
Urban town	1		1		1		1	
Densely populated municipality	1.05 (0.75, 1.47)	0.77	1.09 (0.78, 1.52)	0.63	0.93 (0.65, 1.32)	0.69	0.91 (0.63, 1.31)	0.61
Rural municipality	1.24 (0.98, 1.58)	0.07	1.26 (0.98, 1.61)	0.07	1.17 (0.91, 1.51)	0.22	1.16 (0.90, 1.50)	0.26
**≥ 10 h vs. 7 h**								
Urban town	1		1		1		1	
Densely populated municipality	1.47 (1.02, 2.12)	0.04	1.55 (1.06, 2.28)	0.02	1.53 (1.01, 2.29)	0.04	1.59 (1.06, 2.40)	0.02
Rural municipality	1.64 (1.09, 2.47)	0.02	1.74 (1.17, 2.59)	0.006	1.45 (0.96, 2.20)	0.08	1.45 (0.95, 2.20)	0.08
**Global P-value**	0.06		0.03		0.09		0.03	
**n**	5578		5568		5307		5300	

**Table 4:** Sleep duration: associations with socioeconomic factors

	**Model 1 Adjusted for age and gender**		**Model 2 Adjusted for age, gender, marital status, children and area**		**Model 3 Adjusted for age, gender, marital status, children, area, education, parental education, employment status and income**		**Model 4 Adjusted for age, gender, marital status, children, area, education, parental education, employment status, income and self-perceived health**	
	**OR (95% CI)**	**P-value**	**OR (95% CI)**	**P-value**	**OR (95% CI)**	**P-value**	**OR (95% CI)**	**P-value**
**PARENTAL EDUCATION**								
**< 5 h vs. 7 h**								
High	1		1		1		1	
Intermediate	1.05 (0.38, 2.86)	0.93	1.05 (0.38, 2.89)	0.93	0.89 (0.32, 2.46)	0.82	0.90 (0.32, 2.52)	0.84
Low	1.52 (0.71, 3.24)	0.28	1.44 (0.67, 3.09)	0.35	0.93 (0.41, 2.11)	0.87	0.91 (0.40, 2.07)	0.81
Do not know	1.96 (0.62, 6.20)	0.25	1.79 (0.56, 5.74)	0.33	0.93 (0.26, 3.36)	0.91	0.90 (0.23, 3.43)	0.87
**5 h vs. 7 h**								
High	1		1		1		1	
Intermediate	5.27 (1.20, 23.2)	0.03	5.31 (1.21, 23.3)	0.03	4.47 (1.02, 19.6)	0.05	4.51 (1.09, 18.7)	0.04
Low	3.84 (0.94, 15.6)	0.06	3.61 (0.98, 14.7)	0.07	2.36 (0.57, 9.76)	0.24	2.31 (0.59, 9.12)	0.23
Do not know	4.73 (0.82, 27.4)	0.08	4.36 (0.76, 25.1)	0.10	2.56 (0.43, 15.3)	0.30	2.54 (0.41, 15.7)	0.31
**6 h vs. 7 h**								
High	1		1		1		1	
Intermediate	0.87 (0.53, 1.44)	0.60	0.88 (0.53, 1.46)	0.63	0.87 (0.52, 1.44)	0.59	0.87 (0.53, 1.45)	0.60
Low	0.99 (0.68, 1.43)	0.95	0.98 (0.68, 1.42)	0.92	0.90 (0.60, 1.33)	0.59	0.89 (0.60, 1.33)	0.58
Do not know	0.53 (0.21, 1.29)	0.16	0.48 (0.19, 1.19)	0.12	0.44 (0.17, 1.11)	0.08	0.44 (0.17, 1.11)	0.08
**8 h vs. 7 h**								
High	1		1		1		1	
Intermediate	1.00 (0.71, 1.40)	1.00	0.99 (0.71, 1.39)	0.96	0.97 (0.69, 1.35)	0.84	0.98 (0.70, 1.37)	0.90
Low	0.96 (0.74, 1.26)	0.79	0.94 (0.72, 1.22)	0.65	0.94 (0.72, 1.22)	0.63	0.94 (0.72, 1.24)	0.68
Do not know	0.93 (0.57, 1.51)	0.76	0.91 (0.56, 1.49)	0.71	0.81 (0.47, 1.39)	0.45	0.82 (0.48, 1.41)	0.47
**9 h vs. 7 h**								
High	1		1		1		1	
Intermediate	0.84 (0.52, 1.38)	0.50	0.85 (0.52, 1.39)	0.51	0.80 (0.48, 1.33)	0.38	0.81 (0.48, 1.35)	0.41
Low	0.82 (0.55, 1.22)	0.33	0.82 (0.54, 1.23)	0.33	0.77 (0.50, 1.18)	0.23	0.77 (0.50, 1.19)	0.24
Do not know	0.81 (0.33, 1.98)	0.65	0.67 (0.26, 1.70)	0.40	0.36 (0.12, 1.08)	0.07	0.36 (0.12, 1.08)	0.07
**≥ 10 h vs. 7 h**								
High	1		1		1		1	
Intermediate	2.76 (0.91, 8.37)	0.07	2.63 (0.87, 7.93)	0.08	2.19 (0.72, 6.68)	0.17	2.37 (0.76, 7.35)	0.14
Low	1.88 (0.70, 5.00)	0.21	1.58 (0.60, 4.18)	0.35	1.11 (0.41, 3.02)	0.84	1.15 (0.42, 3.17)	0.78
Do not know	0.57 (0.06, 5.47)	0.63	-	-	-	-	-	-
**Global P-value**	0.44		<0.0001		<0.0001		<0.0001	
**n**	5445		5437		5307		5300	
**OWN EDUCATION**								
**< 5 h vs. 7 h**								
high (> 12)	1		1		1		1	
medium (10–12)	1.86 (1.21, 2.86)	0.005	1.78 (1.14, 2.78)	0.01	1.67 (1.02, 2.73)	0.04	1.60 (0.97, 2.64)	0.07
low (7–9)	2.31 (1.46, 3.64)	0.0003	2.17 (1.36, 3.48)	0.001	2.03 (1.19, 3.46)	0.009	1.84 (1.07, 3.16)	0.03
very low (< 7)	2.55 (1.46, 4.44)	0.001	2.25 (1.27, 3.97)	0.005	1.53 (0.79, 2.98)	0.21	1.27 (0.64, 2.53)	0.50
**5 h vs. 7 h**								
high (> 12)	1		1		1		1	
medium (10–12)	2.37 (1.44, 3.91)	0.0007	2.25 (1.36, 3.71)	0.002	2.11 (1.22, 3.63)	0.007	1.97 (1.15, 3.38)	0.01
low (7–9)	2.27 (1.36, 3.79)	0.002	2.10 (1.24, 3.56)	0.006	1.79 (0.97, 3.31)	0.06	1.54 (0.84, 2.82)	0.16
very low (< 7)	2.29 (1.14, 4.60)	0.02	2.02 (1.03, 3.97)	0.04	1.54 (0.73, 3.24)	0.25	1.27 (0.60, 2.69)	0.53
**6 h vs. 7 h**								
high (> 12)	1		1		1		1	
medium (10–12)	1.10 (0.87, 1.38)	0.42	1.07 (0.85, 1.35)	0.56	1.03 (0.81, 1.33)	0.79	1.02 (0.79, 1.31)	0.88
low (7–9)	1.29 (0.99, 1.68)	0.06	1.26 (0.96, 1.63)	0.09	1.23 (0.91, 1.66)	0.17	1.20 (0.88, 1.62)	0.24
very low (< 7)	1.61 (1.06, 2.43)	0.02	1.54 (1.01, 2.34)	0.05	1.39 (0.87, 2.23)	0.17	1.34 (0.83, 2.15)	0.23
**8 h vs. 7 h**								
high (> 12)	1		1		1		1	
medium (10–12)	0.89 (0.75, 1.07)	0.21	0.89 (0.74, 1.06)	0.18	0.85 (0.71, 1.02)	0.09	0.85 (0.71, 1.02)	0.08
low (7–9)	1.01 (0.83, 1.22)	0.95	0.98 (0.81, 1.19)	0.83	0.92 (0.74, 1.15)	0.46	0.92 (0.74, 1.14)	0.44
very low (< 7)	1.07 (0.78, 1.47)	0.68	1.01 (0.73, 1.42)	0.93	0.86 (0.59, 1.27)	0.45	0.85 (0.57, 1.25)	0.40
**9 h vs. 7 h**								
high (> 12)	1		1		1		1	
medium (10–12)	0.85 (0.65, 1.11)	0.23	0.85 (0.64, 1.11)	0.23	0.87 (0.64, 1.18)	0.37	0.86 (0.64, 1.16)	0.33
low (7–9)	1.09 (0.81, 1.46)	0.57	1.05 (0.78, 1.40)	0.76	0.92 (0.66, 1.28)	0.62	0.91 (0.65, 1.27)	0.57
very low (< 7)	1.46 (0.82, 2.60)	0.20	1.31 (0.73, 2.35)	0.37	0.92 (0.50, 1.72)	0.80	0.90 (0.48, 1.67)	0.73
**≥ 10 h vs. 7 h**								
high (> 12)	1		1		1		1	
medium (10–12)	1.55 (0.96, 2.51)	0.07	1.42 (0.87, 2.31)	0.16	1.26 (0.72, 2.20)	0.43	1.19 (0.68, 2.10)	0.55
low (7–9)	1.84 (1.17, 2.92)	0.009	1.54 (0.98, 2.44)	0.06	1.03 (0.56, 1.90)	0.93	0.90 (0.48, 1.69)	0.74
very low (< 7)	2.69 (1.46, 4.96)	0.002	2.19 (1.15, 4.15)	0.02	1.41 (0.65, 3.02)	0.38	1.18 (0.53, 2.61)	0.68
**Global P-value**	<0.0001		0.0003		0.03		0.05	
**n**	5560		5550		5307		5300	
**HOUSEHOLD INCOME LEVEL**								
**< 5 h vs. 7 h**								
Highest income quartile	1		1		1		1	
3^rd^	1.28 (0.87, 1.86)	0.21	1.38 (0.93, 2.05)	0.11	1.25 (0.81, 1.92)	0.32	1.26 (0.81, 1.95)	0.30
2^nd^	1.63 (1.09, 2.42)	0.02	1.68 (1.13, 2.51)	0.01	1.33 (0.81, 2.18)	0.26	1.27 (0.77, 2.10)	0.34
lowest income quartile	1.87 (1.27, 2.77)	0.002	1.74 (1.14, 2.65)	0.01	1.34 (0.81, 2.21)	0.25	1.28 (0.77, 2.12)	0.34
**5 h vs. 7 h**								
Highest income quartile	1		1		1		1	
3^rd^	1.24 (0.72, 2.12)	0.44	1.39 (0.81, 2.38)	0.23	1.11 (0.63, 1.96)	0.72	1.07 (0.61, 1.89)	0.82
2^nd^	1.54 (0.92, 2.58)	0.10	1.63 (0.96, 2.76)	0.07	1.29 (0.73, 2.30)	0.38	1.23 (0.69, 2.19)	0.48
lowest income quartile	2.29 (1.38, 3.81)	0.002	2.25 (1.33, 3.82)	0.003	1.68 (0.91, 3.11)	0.09	1.55 (0.84, 2.87)	0.16
**6 h vs. 7 h**								
Highest income quartile	1		1		1		1	
3^rd^	1.17 (0.90, 1.52)	0.24	1.25 (0.94, 1.65)	0.12	1.21 (0.91, 1.61)	0.19	1.20 (0.90, 1.59)	0.21
2^nd^	1.29 (1.00, 1.65)	0.05	1.34 (1.03, 1.75)	0.03	1.25 (0.93, 1.67)	0.13	1.23 (0.92, 1.64)	0.17
lowest income quartile	1.56 (1.20, 2.02)	0.0008	1.50 (1.13, 1.99)	0.005	1.39 (0.98, 1.97)	0.06	1.36 (0.96, 1.91)	0.08
**8 h vs. 7 h**								
Highest income quartile	1		1		1		1	
3^rd^	1.19 (1.01, 1.40)	0.04	1.21 (1.03, 1.44)	0.02	1.21 (1.01, 1.44)	0.04	1.20 (1.01, 1.44)	0.04
2^nd^	1.18 (0.99, 1.41)	0.07	1.20 (1.01, 1.44)	0.04	1.17 (0.96, 1.43)	0.12	1.17 (0.96, 1.43)	0.12
lowest income quartile	1.31 (1.07, 1.60)	0.009	1.36 (1.10, 1.68)	0.005	1.32 (1.03, 1.70)	0.03	1.31 (1.02, 1.68)	0.04
**9 h vs. 7 h**								
Highest income quartile	1		1		1		1	
3^rd^	1.56 (1.16, 2.11)	0.004	1.66 (1.23, 2.24)	0.0009	1.47 (1.07, 2.00)	0.02	1.47 (1.08, 2.01)	0.01
2^nd^	1.59 (1.15, 2.19)	0.005	1.67 (1.21, 2.32)	0.002	1.20 (0.80, 1.78)	0.38	1.19 (0.80, 1.77)	0.40
lowest income quartile	2.31 (1.69, 3.15)	<0.0001	2.47 (1.77, 3.44)	<0.0001	1.67 (1.11, 2.50)	0.01	1.65 (1.10, 2.48)	0.01
**≥ 10 h vs. 7 h**								
Highest income quartile	1		1		1		1	
3^rd^	1.41 (0.76, 2.61)	0.27	1.72 (0.92, 3.20)	0.09	1.16 (0.61, 2.20)	0.65	1.13 (0.60, 2.12)	0.71
2^nd^	3.17 (1.84, 5.46)	<0.0001	3.66 (2.09, 6.40)	<0.0001	1.75 (0.96, 3.20)	0.07	1.60 (0.88, 2.92)	0.12
lowest income quartile	5.02 (2.83, 8.89)	<0.0001	5.29 (2.90, 9.65)	<0.0001	2.20 (1.12, 4.32)	0.02	1.99 (1.02, 3.87)	0.04
**Global P-value**	<0.0001		<0.0001		0.20		0.34	
**n**	5453		5453		5307		5300	
**EMPLOYMENT STATUS**								
**< 5 h vs. 7 h**								
Working	1		1		1		1	
Unemployed	1.82 (1.07, 3.09)	0.03	1.65 (0.97, 2.81)	0.06	1.34 (0.75, 2.39)	0.32	1.15 (0.65, 2.04)	0.63
Disability pension	2.02 (1.16, 3.51)	0.01	1.92 (1.10, 3.34)	0.02	1.53 (0.81, 2.89)	0.19	1.17 (0.62, 2.20)	0.62
Old age retirement	1.29 (0.74, 2.23)	0.37	1.29 (0.74, 2.24)	0.36	1.04 (0.57, 1.91)	0.89	1.01 (0.54, 1.88)	0.98
Other pension	1.28 (0.61, 2.70)	0.52	1.21 (0.57, 2.55)	0.62	1.07 (0.49, 2.33)	0.86	1.06 (0.48, 2.37)	0.88
Other	1.23 (0.43, 3.51)	0.69	1.25 (0.43, 3.60)	0.69	1.00 (0.30, 3.29)	1.00	0.93 (0.28, 3.12)	0.90
**5 h vs. 7 h**								
Working	1		1		1		1	
Unemployed	1.76 (0.93, 3.35)	0.08	1.64 (0.87, 3.09)	0.13	1.21 (0.63, 2.31)	0.57	0.99 (0.52, 1.89)	0.98
Disability pension	2.54 (1.32, 4.88)	0.005	2.52 (1.31, 4.82)	0.005	2.08 (1.07, 4.03)	0.03	1.40 (0.71, 2.77)	0.33
Old age retirement	1.37 (0.70, 2.70)	0.36	1.42 (0.72, 2.80)	0.31	1.13 (0.56, 2.28)	0.74	0.97 (0.47, 1.99)	0.94
Other pension	0.46 (0.11, 1.87)	0.27	0.44 (0.10, 1.86)	0.26	0.37 (0.09, 1.51)	0.17	0.36 (0.09, 1.47)	0.15
Other	1.29 (0.35, 4.68)	0.70	1.44 (0.40, 5.26)	0.58	1.40 (0.39, 5.05)	0.61	1.25 (0.39, 4.02)	0.71
**6 h vs. 7 h**								
Working	1		1		1		1	
Unemployed	1.09 (0.74, 1.60)	0.66	0.99 (0.67, 1.46)	0.94	0.85 (0.56, 1.30)	0.46	0.84 (0.55, 1.28)	0.42
Disability pension	1.50 (1.05, 2.15)	0.03	1.39 (0.96, 1.99)	0.08	1.12 (0.77, 1.61)	0.55	1.04 (0.72, 1.52)	0.82
Old age retirement	1.22 (0.89, 1.68)	0.22	1.18 (0.85, 1.65)	0.32	0.97 (0.67, 1.39)	0.86	0.97 (0.68, 1.40)	0.89
Other pension	0.68 (0.35, 1.31)	0.25	0.65 (0.33, 1.27)	0.21	0.62 (0.31, 1.24)	0.18	0.62 (0.31, 1.24)	0.18
Other	1.13 (0.64, 2.00)	0.67	1.20 (0.68, 2.13)	0.53	1.08 (0.59, 1.97)	0.80	1.07 (0.59, 1.94)	0.82
**8 h vs. 7 h**								
Working	1		1		1		1	
Unemployed	1.17 (0.93, 1.48)	0.18	1.17 (0.93, 1.47)	0.19	1.02 (0.79, 1.31)	0.89	1.02 (0.79, 1.31)	0.90
Disability pension	1.40 (1.06, 1.85)	0.02	1.41 (1.06, 1.86)	0.02	1.31 (0.97, 1.77)	0.08	1.31 (0.96, 1.79)	0.09
Old age retirement	1.55 (1.16, 2.07)	0.003	1.60 (1.21, 2.14)	0.001	1.45 (1.08, 1.94)	0.01	1.45 (1.09, 1.94)	0.01
Other pension	1.21 (0.75, 1.96)	0.43	1.20 (0.75, 1.93)	0.45	1.12 (0.69, 1.81)	0.65	1.12 (0.69, 1.82)	0.64
Other	1.69 (1.16, 2.45)	0.006	1.68 (1.15, 2.46)	0.007	1.60 (1.07, 2.37)	0.02	1.61 (1.08, 2.39)	0.02
**9 h vs. 7 h**								
Working	1		1		1		1	
Unemployed	2.19 (1.49, 3.21)	0.0001	2.10 (1.43, 3.09)	0.0002	1.90 (1.23, 2.92)	0.003	1.87 (1.22, 2.87)	0.004
Disability pension	4.24 (2.55, 7.05)	<0.0001	4.07 (2.44, 6.79)	<0.0001	3.85 (2.29, 6.48)	<0.0001	3.62 (2.13, 6.15)	<0.0001
Old age retirement	5.62 (3.33, 9.48)	<0.0001	5.61 (3.34, 9.43)	<0.0001	5.49 (3.11, 9.67)	<0.0001	5.46 (3.11, 9.60)	<0.0001
Other pension	2.75 (1.32, 5.72)	0.007	2.38 (1.14, 4.95)	0.02	2.27 (1.06, 4.88)	0.03	2.24 (1.04, 4.83)	0.04
Other	3.40 (2.28, 5.09)	<0.0001	3.34 (2.20, 5.06)	<0.0001	3.09 (2.02, 4.74)	<0.0001	3.08 (2.00, 4.74)	<0.0001
**≥ 10 h vs. 7 h**								
Working	1		1		1		1	
Unemployed	7.22 (4.00, 13.0)	<0.0001	6.11 (3.39, 11.0)	<0.0001	4.60 (2.45, 8.64)	<0.0001	4.10 (2.19, 7.67)	<0.0001
Disability pension	17.3 (9.29, 32.3)	<0.0001	15.2 (8.16, 28.2)	<0.0001	12.5 (6.51, 24.2)	<0.0001	9.53 (4.85, 18.7)	<0.0001
Old age retirement	10.3 (5.24, 20.3)	<0.0001	11.1 (5.57, 22.2)	<0.0001	9.21 (4.43, 19.2)	<0.0001	9.23 (4.34, 19.6)	<0.0001
Other pension	5.31 (1.82, 15.5)	0.002	3.92 (1.16, 13.3)	0.03	3.80 (1.09, 13.3)	0.04	3.70 (1.07, 12.8)	0.04
Other	9.14 (4.51, 18.5)	<0.0001	11.4 (5.75, 22.7)	<0.0001	8.58 (3.88, 19.0)	<0.0001	8.27 (3.78, 18.1)	<0.0001
**Global P-value**	<0.0001		<0.0001		<0.0001		<0.0001	
**n**	5578		5568		5307		5300	

**Table 5:** Insomnia-related symptoms: associations with sociodemographic factors

	**Model 1 Adjusted for age and gender**		**Model 2 Adjusted for age, gender, marital status, children and area**		**Model 3 Adjusted for age, gender, marital status, children, area, education, parental education, employment status and income**		**Model 4 Adjusted for age, gender, marital status, children, area, education, parental education, employment status, income and self-perceived health**	
	**OR (95% CI)**	**P-value**	**OR (95% CI)**	**P-value**	**OR (95% CI)**	**P-value**	**OR (95% CI)**	**P-value**
**MARITAL STATUS**								
**Occasional symptoms vs. Good sleepers**								
Married/living together	1		1		1		1	
Single	1.23 (1.01, 1.51)	0.04	1.12 (0.91, 1.39)	0.28	1.06 (0.84, 1.33)	0.63	1.05 (0.84, 1.32)	0.66
Divorced/separated	1.13 (0.90, 1.42)	0.29	1.08 (0.86, 1.36)	0.53	0.99 (0.78, 1.25)	0.92	0.97 (0.76, 1.23)	0.81
Widowed	1.50 (1.13, 1.98)	0.005	1.52 (1.14, 2.01)	0.004	1.50 (1.14, 1.98)	0.004	1.54 (1.17, 2.03)	0.002
**Frequent symptoms vs. Good sleepers**								
Married/living together	1		1		1		1	
Single	1.08 (0.80, 1.44)	0.62	0.88 (0.65, 1.21)	0.44	0.75 (0.54, 1.04)	0.08	0.75 (0.55, 1.04)	0.08
Divorced/separated	1.60 (1.20, 2.12)	0.001	1.46 (1.10, 1.91)	0.008	1.17 (0.87, 1.56)	0.30	1.06 (0.78, 1.45)	0.71
Widowed	1.33 (1.00, 1.77)	0.05	1.38 (1.04, 1.83)	0.03	1.20 (0.88, 1.63)	0.26	1.24 (0.89, 1.72)	0.20
**Global P-value**	0.0004		0.0006		0.02		0.02	
**n**	5578		5568		5307		5300	
**NUMBER OF CHILDREN**								
**Occasional symptoms vs. Good sleepers**								
No under 18 years old children	1		1		1		1	
Under 7-year-olds	0.87 (0.65, 1.16)	0.35	0.89 (0.66, 1.19)	0.42	0.86 (0.61, 1.19)	0.36	0.89 (0.63, 1.24)	0.48
7–17-year-olds	0.80 (0.66, 0.96)	0.02	0.82 (0.67, 1.00)	0.05	0.79 (0.64, 0.97)	0.02	0.79 (0.64, 0.97)	0.03
Both	0.73 (0.53, 0.99)	0.04	0.74 (0.54, 1.03)	0.07	0.64 (0.45, 0.91)	0.01	0.68 (0.47, 0.97)	0.04
**Frequent symptoms vs. Good sleepers**								
No under 18 years old children	1		1		1		1	
Under 7-year-olds	0.31 (0.18, 0.53)	<0.0001	0.31 (0.18, 0.53)	<0.0001	0.27 (0.15, 0.48)	<0.0001	0.30 (0.17, 0.54)	0.0001
7–17-year-olds	0.68 (0.54, 0.86)	0.002	0.67 (0.52, 0.87)	0.002	0.65 (0.50, 0.86)	0.002	0.67 (0.51, 0.89)	0.005
Both	0.51 (0.34, 0.77)	0.001	0.50 (0.33, 0.77)	0.002	0.42 (0.27, 0.66)	0.0002	0.49 (0.31, 0.78)	0.003
**Global P-value**	<0.0001		0.0001		<0.0001		0.0002	
**n**	5568		5568		5307		5300	
**RESIDENTIAL AREA**								
**Occasional symptoms vs. Good sleepers**								
Urban town	1		1		1		1	
Densely populated municipality	0.97 (0.83, 1.15)	0.75	0.99 (0.83, 1.16)	0.86	0.99 (0.84, 1.17)	0.88	0.99 (0.84, 1.17)	0.94
Rural municipality	0.97 (0.81, 1.15)	0.69	0.99 (0.83, 1.17)	0.89	0.95 (0.79, 1.15)	0.62	0.92 (0.77, 1.11)	0.39
**Frequent symptoms vs. Good sleepers**								
Urban town	1		1		1		1	
Densely populated municipality	0.92 (0.74, 1.15)	0.47	0.96 (0.77, 1.19)	0.69	0.86 (0.68, 1.09)	0.22	0.92 (0.71, 1.19)	0.53
Rural municipality	0.99 (0.82, 1.18)	0.88	1.03 (0.86, 1.23)	0.75	0.96 (0.79, 1.17)	0.71	0.94 (0.75, 1.17)	0.59
**Global P-value**	0.94		0.98		0.77		0.87	
**n**	5578		5568		5307		5300	

**Table 6:** Insomnia-related symptoms: associations with socioeconomic factors

	**Model 1 Adjusted for age and gender**		**Model 2 Adjusted for age, gender, marital status, children and area**		**Model 3 Adjusted for age, gender, marital status, children, area, education, parental education, employment status and income**		**Model 4 Adjusted for age, gender, marital status, children, area, education, parental education, employment status, income and self-perceived health**	
	**OR (95% CI)**	**P-value**	**OR (95% CI)**	**P-value**	**OR (95% CI)**	**P-value**	**OR (95% CI)**	**P-value**
**PARENTAL EDUCATION**								
**Occasional symptoms vs. Good sleepers**								
High	1		1		1		1	
Intermediate	0.90 (0.62, 1.32)	0.60	0.90 (0.62, 1.31)	0.59	0.89 (0.60, 1.30)	0.53	0.90 (0.61, 1.33)	0.59
Low	1.13 (0.84, 1.53)	0.41	1.13 (0.83, 1.52)	0.44	1.06 (0.77, 1.45)	0.72	1.03 (0.75, 1.41)	0.87
Do not know	0.52 (0.26, 1.06)	0.07	0.52 (0.25, 1.05)	0.07	0.54 (0.26, 1.13)	0.10	0.57 (0.27, 1.18)	0.13
**Frequent symptoms vs. Good sleepers**								
High	1		1		1		1	
Intermediate	1.09 (0.68, 1.73)	0.72	1.10 (0.69, 1.75)	0.70	1.00 (0.62, 1.61)	1.00	1.04 (0.63, 1.72)	0.88
Low	1.07 (0.73, 1.56)	0.73	1.03 (0.70, 1.51)	0.88	0.82 (0.55, 1.24)	0.35	0.80 (0.52, 1.23)	0.31
Do not know	1.43 (0.77, 2.63)	0.25	1.40 (0.76, 2.61)	0.28	1.01 (0.52, 1.96)	0.97	1.11 (0.52, 2.39)	0.78
**Global P-value**	0.08		0.08		0.16		0.15	
**n**	5445		5437		5307		5300	
**OWN EDUCATION**								
**Occasional symptoms vs. Good sleepers**								
high (> 12)	1		1		1		1	
medium (10-12)	1.06 (0.89, 1.26)	0.54	1.05 (0.88, 1.25)	0.59	0.99 (0.83, 1.18)	0.89	0.94 (0.78, 1.12)	0.47
low (7–9)	1.15 (0.96, 1.38)	0.12	1.13 (0.94, 1.35)	0.19	1.03 (0.84, 1.24)	0.80	0.92 (0.76, 1.12)	0.42
very low (< 7)	0.97 (0.73, 1.30)	0.83	0.94 (0.70, 1.25)	0.65	0.78 (0.57, 1.06)	0.11	0.69 (0.50, 0.95)	0.02
**Frequent symptoms vs. Good sleepers**								
high (> 12)	1		1		1		1	
medium (10–12)	1.42 (1.12, 1.80)	0.004	1.34 (1.06, 1.71)	0.02	1.27 (0.96, 1.67)	0.09	1.14 (0.86, 1.51)	0.36
low (7–9)	1.56 (1.22, 1.99)	0.0004	1.48 (1.15, 1.89)	0.002	1.19 (0.89, 1.58)	0.24	0.93 (0.69, 1.26)	0.63
very low (< 7)	1.26 (0.88, 1.80)	0.20	1.30 (0.89, 1.90)	0.17	1.03 (0.68, 1.56)	0.90	0.75 (0.47, 1.17)	0.20
**Global P-value**	0.01		0.07		0.33		0.19	
**n**	5560		5550		5307		5300	
**HOUSEHOLD INCOME LEVEL**								
**Occasional symptoms vs. Good sleepers**								
Highest income quartile	1		1		1		1	
3^rd^	0.95 (0.79, 1.14)	0.58	1.02 (0.84, 1.24)	0.82	1.02 (0.84, 1.23)	0.83	0.99 (0.82, 1.20)	0.92
2^nd^	1.22 (1.02, 1.47)	0.03	1.33 (1.10, 1.61)	0.004	1.28 (1.05, 1.56)	0.01	1.21 (0.98, 1.48)	0.07
lowest income quartile	1.27 (1.04, 1.54)	0.02	1.36 (1.11, 1.66)	0.003	1.27 (1.02, 1.58)	0.03	1.18 (0.95, 1.47)	0.13
**Frequent symptoms vs. Good sleepers**								
Highest income quartile	1		1		1		1	
3^rd^	1.03 (0.80, 1.33)	0.83	1.23 (0.93, 1.62)	0.15	1.03 (0.77, 1.38)	0.83	1.01 (0.75, 1.36)	0.96
2^nd^	1.48 (1.16, 1.88)	0.002	1.77 (1.37, 2.28)	<0.0001	1.33 (1.00, 1.77)	0.05	1.19 (0.88, 1.59)	0.25
lowest income quartile	1.89 (1.46, 2.45)	<0.0001	2.32 (1.77, 3.04)	<0.0001	1.54 (1.13, 2.09)	0.006	1.35 (0.98, 1.86)	0.06
**Global P-value**	<0.0001		<0.0001		0.006		0.15	
**n**	5453		5453		5307		5300	
**EMPLOYMENT STATUS**								
**Occasional symptoms vs. Good sleepers**								
Working	1		1		1		1	
Unemployed	1.49 (1.17, 1.91)	0.001	1.44 (1.13, 1.83)	0.004	1.25 (0.96, 1.62)	0.10	1.16 (0.89, 1.52)	0.27
Disability pension	1.34 (1.00, 1.79)	0.05	1.29 (0.96, 1.73)	0.09	1.19 (0.88, 1.61)	0.26	0.96 (0.71, 1.30)	0.80
Old age retirement	0.97 (0.73, 1.28)	0.81	0.93 (0.70, 1.24)	0.62	0.84 (0.62, 1.13)	0.24	0.86 (0.64, 1.16)	0.32
Other pension	0.99 (0.65, 1.49)	0.94	0.93 (0.61, 1.41)	0.72	0.82 (0.53, 1.28)	0.39	0.83 (0.54, 1.28)	0.39
Other	1.54 (1.07, 2.20)	0.02	1.58 (1.09, 2.29)	0.02	1.32 (0.89, 1.95)	0.16	1.31 (0.89, 1.93)	0.17
**Frequent symptoms vs. Good sleepers**								
Working	1		1		1		1	
Unemployed	3.19 (2.42, 4.21)	<0.0001	3.02 (2.29, 3.98)	<0.0001	2.48 (1.81, 3.38)	<0.0001	2.00 (1.45, 2.76)	<0.0001
Disability pension	3.30 (2.34, 4.67)	<0.0001	3.36 (2.38, 4.73)	<0.0001	2.88 (1.99, 4.17)	<0.0001	1.69 (1.14, 2.50)	0.009
Old age retirement	1.15 (0.79, 1.69)	0.46	1.31 (0.89, 1.93)	0.17	1.17 (0.78, 1.74)	0.45	1.12 (0.74, 1.68)	0.59
Other pension	1.40 (0.82, 2.39)	0.22	1.44 (0.85, 2.45)	0.18	1.30 (0.74, 2.28)	0.36	1.26 (0.71, 2.23)	0.43
Other	1.69 (1.04, 2.75)	0.03	2.10 (1.29, 3.42)	0.003	1.61 (0.94, 2.76)	0.08	1.47 (0.87, 2.49)	0.15
**Global P-value**	<0.0001		<0.0001		<0.0001		0.0004	
**n**	5578		5568		5307		5300	

All models were cumulative. Model 1 included only gender and age. Sociodemographic variables (marital status, number of children, residential area) were added in model 2, and socioeconomic variables were added in model 3. Final model 4 included also self-perceived health. In addition to 95% CI, *p*-values for categories and global *p*-values for the indicators (Wald F) are reported in the Tables. As there were no gender interactions (the associations of the examined sociodemographic and socioeconomic factors with sleep duration and insomnia-related symptoms were similar for both women and men), all analyses were conducted in the pooled data adjusting for gender. The analyses were conducted using SAS version 9.2 and SUDAAN.

## Results

### Descriptive analyses

Around 70% of women and men slept for an average 7-8 hours a day (Table 1). Short (≤6 h) sleep was more prevalent than long (≥9 h) sleep, and women tended to report longer sleep duration than men. The prevalence of both short and very long sleep duration tended to increase with age, whereas those living with a partner were less likely to report short sleep than divorced or separated persons. Participants with low education tended to sleep less than those with high education. Of participants on disability retirement, 9% slept ≥10 hours, while the corresponding prevalence was 5% among those on an old age pension and 1% among employed participants. Very short sleep (<5 h), short sleep (≤ 5 h), and long sleep (≥9 h) were more prevalent among those with poorer self-perceived health.

Women, older, divorced, or separated participants reported more frequent insomnia-related symptoms (Table 2). The prevalence of frequent insomnia-related symptoms was only 5% for those with small children (under 7 years of age) as compared to 14% for those without children. The highest prevalence of frequent insomnia-related symptoms was found among unemployed participants (23%) and disability retirees (24%), whereas among working participants it was less than 10%. Insomnia-related symptoms were more common among participants with poorer self-perceived health.

### Sociodemographic and socioeconomic differences in sleep duration and insomnia-related symptoms

#### Sleep duration

Associations between sleep duration and sociodemographic factors broadly followed a U-shaped pattern (Table 3). Single participants had higher odds of sleeping for <5 hours (*OR* 1.74, 95% *CI* 1.15-2.64), 6 hours (*OR* 1.52, 95% *CI* 1.13-2.05) or ≥10 hours (*OR* 2.33, 95% *CI* 1.51–3.58) as compared to those who were married (Table 3, Model 1). Divorced and separated participants also had higher odds to sleep <5 hours (*OR* 1.76, 95% *CI* 1.13–2.72), 5 hours (*OR* 1.82, 95% *CI* 1.11–2.97), 6 hours (*OR* 1.73, 95% *CI* 1.34–2.23), or ≥10 hours (*OR* 1.66, 95% *CI* 0.99–2.77) as compared to married participants. Similarly to divorced and separated, widowed had higher odds of reporting short sleep (5 hours and 6 hours). Participants with 7–17-year-old children were less likely to sleep ≥10 hours (*OR* 0.59, 95% *CI* 0.35–0.99) as compared to those who did not have children. Participants living in densely populated municipalities (*OR* 1.47, 95% *CI* 1.02–2.12) or rural municipalities (*OR* 1.64, 95% *CI* 1.09–2.47) had, in turn, higher odds of sleeping for ≥10 hours as compared to participants from urban towns.

U-shaped associations were also observed between sleep duration and socioeconomic circumstances, with those in lower socioeconomic positions reporting both more short sleep and long sleep (Table 4). First, those with intermediate parental education had higher odds of sleeping for 5 hours (*OR* 5.27, 95% *CI* 1.20–23.20) as compared to those whose parents’ had had high education (Table 4, Model 1). Similar patterns were suggested for those with low parental education. Participants with low own education also had higher odds of short and long sleep as compared to those with high education. Concerning sleep duration of <5 hours and 5 hours, all educational groups differed from the high education group. Income level was also consistently associated with both short and long sleep duration. The associations were the strongest concerning long sleepers. Those with low (*OR* 3.17, 95% *CI* 1.84–5.46) or very low (*OR* 5.02, 95% *CI* 2.83–8.89) household income levels had higher odds of reporting sleeping for ≥10 hours as compared to those with the highest household income levels. Similar patterns, albeit weaker estimates, were observed for 8 and 9 hour sleepers. Additionally, those with the lowest incomes had higher odds of sleeping for <5 hours (*OR* 1.87, 95% *CI* 1.27–2.77), 5 hours (*OR* 2.29, 95% *CI* 1.38–3.81), and 6 hours (*OR* 1.56, 95% *CI* 1.20–2.02). Employment status had the strongest associations with long sleep duration. The unemployed (*OR* 7.22, 95% *CI* 4.00–13.00) and those who had retired due to old age (*OR* 10.30, 95% *CI* 5.24–20.30) or disability (*OR* 17.30, 95% *CI* 9.29–32.30) had particularly high odds of reporting sleeping for ≥10 hours as compared to the employed participants. However, as the groups were small, the confidence intervals were very wide. Similar differences among employed, unemployed, and retired participants were also found for 8 and 9 hour sleepers, but the effect sizes were weaker. Additionally, disability retirees had higher odds of sleeping for <5 hours (*OR* 2.02, 95% *CI* 1.16–3.51) and 5 hours (*OR* 2.54, 95% *CI* 1.32–4.88) as compared to employed participants. Similar patterns were found for the unemployed.

Associations largely remained after all sociodemographic factors were included in the Model 2 (Tables 3 and 4). After additionally adjusting for socioeconomic circumstances (Tables 3 and 4, Model 3), associations between marital status and long sleep were reduced, but the divorced and separated participants continued to have higher odds of sleeping for < 5 hours and 6 hours, singles 6 hours and ≥10 hours and widowed 5 hours. Those with small children had lower odds of short and long sleep. The adjustments strengthened particularly the association for long sleep (≥10 hours). The associations between low income and short sleep were reduced after adjustments, while low income remained associated with long sleep. Those with the lowest income had higher odds of sleeping for 9 hours and ≥10 hours as compared to those with high income, although the effect size was largely reduced in Model 3. As with income, the associations between employment status and short sleep reduced after simultaneous adjustments for sociodemographic and socioeconomic factors (Table 4, Model 3). However, unemployment remained associated with 5 hour sleep, and both the unemployed and those retired due to disability or old age had higher odds of sleeping for 9 hours or ≥10 hours.

Adjusting for perceived health did not substantially change the observed associations (Tables 3 and 4, Model 4). While the association between short sleep (≤ 5 hours) and disability retirement attenuated, the association between long sleep (9 hours and ≥ 10 hours) and disability retirement was robust to the adjustment.

#### Insomnia-related symptoms

Age and gender adjusted multinomial logistic regression analyses showed that widowed participants had higher odds of reporting both occasional (*OR* 1.50, 95% *CI* 1.13–1.98) and frequent (*OR* 1.33, 95% *CI* 1.00–1.77) insomnia-related symptoms as compared to those who were married (Table 5, Model 1). The divorced and separated participants also reported more often frequent insomnia-related symptoms (*OR* 1.60, 95% *CI* 1.20–2.12) as compared to those who were married. Having under 7-year-old children (*OR* 0.31, 95% *CI* 0.18–0.53) and 7–17 year-old children (*OR* 0.68, 95% *CI* 0.54–0.86) was, in turn, inversely associated with frequent insomnia-related symptoms. Similar pattern was observed for occasional insomnia-related symptoms.

Concerning socioeconomic circumstances, parental education was not associated with insomnia-related symptoms, whereas those participants with low (*OR* 1.56, 95% *CI* 1.22–1.99) and medium (*OR* 1.42, 95% *CI* 1.12–1.80) own education reported more often frequent insomnia-related symptoms than those with high education (Table 6, Model 1). Those with low or very low household income reported more often both frequent and occasional insomnia-related symptoms as compared to those with high income. The associations were the strongest for those with the lowest income (*OR* 1.89, 95% *CI* 1.46–2.45). The most consistent and the strongest associations were found between employment status and insomnia-related symptoms. Unemployed participants reported more often both occasional (*OR* 1.49, 95% *CI* 1.17–1.91) and, in particular, frequent (*OR* 3.19, 95% *CI* 2.42–4.21) insomnia-related symptoms than those who were employed. Furthermore, those who had retired due to disability reported both more occasional (*OR* 1.34, 95% *CI* 1.00–1.79) and frequent (*OR* 3.30, 95% *CI* 2.34–4.67) insomnia-related symptoms as compared to employed participants, whereas old age retirees slept as well as employed participants.

Adjusting for sociodemographic factors (Tables 5 and 6, Model 2) did not substantially change the observed associations. However, after simultaneous adjustment for both sociodemographic and socioeconomic factors (Tables 5 and 6, Model 3) some of the associations were attenuated. For example, marital status was not anymore associated with frequent insomnia-related symptoms. However, those who had been widowed had higher odds of occasional insomnia-related symptoms as compared to married participants and the inverse associations between having children and occasional and frequent insomnia-related symptoms were also observed in Model 3.

After simultaneous adjustment for sociodemographic and socioeconomic factors the associations between participants’ own education and income level and insomnia-related symptoms were somewhat reduced (Table 6, Model 3). Similar patterns applied for employment status.

Adjusting for perceived health had a minor contribution to the associations between sociodemographic and socioeconomic factors with insomnia-related symptoms (Tables 5 and 6, Model 4). However, after the adjustment the association between household income level and frequent insomnia-related symptoms was reduced.

Finally, we conducted sensitivity analyses to consider the effects of hypnotic use and alcohol consumption (grams per week) on the examined associations between sociodemographic and socioeconomic factors and sleep. The adjustments had negligible effects on the associations tested and thus alcohol and hypnotics were omitted from the final tables (data not shown).

## Discussion and conclusions

### Main findings

This study examined the associations of sociodemographic and socioeconomic circumstances with sleep duration and insomnia-related symptoms among nationally representative sample of Finnish adults. The main findings were as follows:

1) Childhood socioeconomic position was mostly unassociated with adult sleep.

2) After full adjustments, the associations between sleep and sociodemographic or socioeconomic factors were attenuated, except for marital status, household income and employment status which remained associated with sleep duration and insomnia-related symptoms. Sleep is shorter and insomnia-related symptoms are more prevalent among the divorced and separated as compared to married adults. However, income and employment status were the most consistent determinants of short and long sleep duration and insomnia-related symptoms. Thus, those with low household income levels, the unemployed, and disability retirees were the most likely to report poor sleep.

3) Having small or adolescent children was associated with better sleep.

4) A clear gradient was observed in many associations regarding occasional and frequent insomnia-related symptoms. Correspondingly, disadvantaged social position was mostly related to short and long sleep duration, and the strength of the association increased toward the extreme ends of the sleep duration distribution.

### Previous studies

Poor sleep in adulthood may reflect childhood circumstances, chronic problems, and adversity. Accordingly, recent studies have shown that several problems including economic difficulties in childhood are associated with adult sleep quality [8] and insomnia-related symptoms [7]. Contrary to our findings, the association between low parental education and difficulties falling asleep remained in an earlier study, when current socioeconomic position as measured by education and occupation had been accounted for [48]. However, no association was found for sleep maintenance. As our measure did not distinguish between different insomnia symptoms, it is difficult to directly compare the findings. However, in line with this study, parental education has not been associated with insomnia-related symptoms [7]. While lower socioeconomic position in childhood has been linked with chronic diseases and mortality on adulthood, [49] most examined diseases such as coronary heart diseases may take decades to develop. Thus, this may explain the lack of an association between childhood socioeconomic position and sleep, as insomnia symptoms can emerge in adulthood, and in addition to changes in health status, they can be attributable to strenuous working conditions, and different life situations, for example. In all, due to the crudeness of our measure on parental education, more comprehensive data about childhood socioeconomic position is needed to confirm its significance to adult sleep. It is also possible that otherwise disadvantaged childhood circumstances could be important to sleep in adults.

One might have assumed that parents of young children have more sleep problems and sleep less. Unexpectedly, we found that having children was associated with fewer insomnia-related symptoms than not having children. The age range examined did not allow us to separate infants from toddlers or older children. Thus it is possible the requested age (under 7-years) was too inclusive and differences between parents of infants and other children remained undetected. Nonetheless, similar inverse association between number of children in a household and frequent insomnia-related symptoms has also been reported in a British cohort [3]. Even though the models were adjusted for age, it is possible that this cannot fully account for the fact that insomnia-related symptoms were rare among younger adults (with children) and much more prevalent among older adults in our cohort as well as in the British cohort [3]. However, similar association was found when we restricted the analyses to younger (30-45 years old) participants (data not shown). Furthermore, it was repeated among both mothers and fathers. Nonetheless, while it is practically evident that infants disrupt especially their mothers’ sleep, teenaged children may cause worry and anxiety by keeping their mothers awake waiting for their children to return home, for example [14]. It is also possible that parents report their sleep as it were without possible disruptions by their children. As our stratified analyses showed differences in sleep between participants with and without children, which were not explained by age or gender, the reasons for these associations require further scrutiny.

In line with previous studies [1-6], sleep duration and insomnia-related symptoms varied according to gender, age, and marital status. Thus, sleep was shorter and insomnia-related symptoms were more prevalent among older adults and among single, divorced, and widowed adults. However, healthy elderly people have been reported to sleep as well as their younger counterparts [50,51]. Thus, sleep duration and insomnia-related symptoms should not only be seen as a function of age; they are likely to be attributable to other causal factors [51]. Overall, these findings highlight the need of increasing our understanding about the importance of sleep for ageing people and those living alone. Additionally, even though the higher prevalence of insomnia-related symptoms among women is in line with previous evidence, the sociodemographic patterning of sleep was similar between genders as judged by the lack of interactions. Since we focused on multiple social determinants of both sleep duration and insomnia-related symptoms more detailed examination of gender differences was both unfeasible and beyond the scope of this study.

Although residential area was not associated with insomnia-related symptoms, those living in densely populated or rural municipalities tended to sleep more often ≥10 hours than those living in urban towns. Studies of residential area and sleep duration are sparse, but this association might be related to different age and occupational structures among residential areas in Finland. For example, people living in rural areas tend to be older, and more often farmers. In line with our findings concerning an association between long sleep duration and living outside urban cities, a previous study reported that both average sleep duration and subjective sleep need were slightly higher among those living in rural areas as compared to those living in urban areas [11].

Despite clear sociodemographic differences in sleep, deviation from population mean sleep duration, and insomnia-related symptoms varied most consistently with socioeconomic circumstances. The associations between poorer sleep and low income were in line with previous evidence, although the associations were particularly strong in our cohort [1,3]. Unemployment has also been shown to be associated with sleep duration and insomnia-related symptoms in several studies [1,2]. However, the associations between sleep and retirement are complex. Thus, although sleep may tend to improve after old age retirement, for example due to the removal of work-related stressors, sleep among disability retirees is poor before and after retirement [52,53]. Accordingly, we found associations only between disability retirement and occasional and frequent insomnia-related symptoms, whereas the sleep quality of old age retirees did not differ from the employed participants. Finally, further material resources, such as housing tenure and economic difficulties, may contribute to poorer sleep and partly account for sleep inequalities according to income and employment status [3,7,54,55]. As data concerning broader material circumstances were unavailable for this study further scrutiny is needed to corroborate these findings.

This study showed that after mutual adjustment, many associations among sociodemographic and socioeconomic circumstances and sleep are attenuated. This suggests that part of the effects of other determinants is mediated through other determinants. Since we were able to include a broad range of sociodemographic variables, the results show which of the associations remain when the effects of other determinants have been taken into account. Thus this wider approach provided a more detailed understanding on the production of socioeconomic inequalities in sleep and highlighted the importance to consider multiple socioeconomic circumstances simultaneously. After adjustments, the results showed the importance of e.g. income and employment status to sleep over and above the effects of other indicators. This examination of multiple socioeconomic circumstances further confirms that the socioeconomic indicators are not interchangeable, but each indicator has a specific nature and reflects particular socioeconomic circumstances across lifecourse [56-59]. Most previous studies have focused on one or a few indicators as determinants of sleep, but our results suggest the importance of considering multiple indicators simultaneously. Among the adult Finnish population, income and employment status are key socioeconomic determinants of sleep, and are likely to explain the associations between low education and poor sleep, for example. Because most of the associations remained after adjustment for health status, this highlights the role of social factors in poor sleep. Adjusting for health might also bias the estimates if sleep, among other factors, mediates the association among sociodemographic and socioeconomic circumstances and poor health, as suggested earlier [3,60]. As our aim was to focus on a range of sociodemographic determinants of both insomnia-related symptoms and sleep duration, inclusion of a full array of explanatory factors was beyond the scope of this study. However, in previous studies [3,7], several potential explanations for the found socioeconomic inequalities in sleep have been considered. For example, the associations between unemployment, low income and poor sleep could be partly accounted for by worries and stress in addition to poorer health status [3]. Education, in turn, reflects health-related values, behaviours, and attitudes which can be assumed to include sleeping habits as well. High educated are assumed to have better knowledge on the means to improve sleep, importance of sleep to health, and they may also more actively seek help and treatment to their insomnia or sleep deprivation [3,61]. Albeit adjusting for such potential explanatory variables has resulted in attenuation of the associations between sociodemographics and sleep, most of the associations remained.

Finally, our statistical models revealed a clear gradient in many associations, which is in line with previous evidence linking the highest morbidity and mortality risks to the extreme ends of sleep duration distribution [35]. Furthermore, health risks indicated by work disability, for example, are higher for frequent as compared to occasional insomnia symptoms [18,19,62]. In order to promote better health and well-being, our results also highlight the importance to focus on the milder insomnia-related symptoms as well as the more serious ones. As such, insomnia symptoms are highly prevalent in the population and early detection and better identification of such symptoms and risk groups could have a notable effect on public health.

### Strengths and limitations

#### Limitations

Several limitations need to be acknowledged. First, this study was cross-sectional, which makes it impossible to infer on causality of observed associations. Two-way associations between sleep and socioeconomic position are conceivable. Disadvantaged position is likely to be a determinant of sleep due to financial strain and related stress for example. It is also possible that poor sleep, as part of a medical condition that is severe enough to affect the global functioning in the long-term, leads to disability retirement [18,62]. However, as social welfare and health services are relatively good in Finland as compared to many other countries, and all population groups have access to health care, consequences of poor sleep do not necessarily imply varying health cost by socioeconomic position as much as in some other countries. Although we took into account health status in this study, the association between sleep and health is complex, and it is difficult to interpret the effects of health adjustments, and separate primary insomnia from comorbid conditions.

Second, we only had a single item measure for insomnia-related symptoms. The item has, however, been shown to have relevant psychometric properties (associations with other sleep-related variables and outcome variables) in several our previous studies [34,40,63-66]. Such single items have also been shown to have important predictive value for various physical and mental health outcomes [33,67,68]. Although our sleep measures were not validated, validity of similar self-reported items has been assessed in several previous studies [69,70]. Since insomnia-related symptoms tend to be persistent [71], a further limitation of this study was that data about childhood sleep duration and sleep-related problems were not included. However, childhood socioeconomic position could be taken into account, and the main focus of this study was on the associations among current sociodemographic and socioeconomic circumstances, sleep duration, and insomnia-related symptoms among the adult Finnish population.

Third, retrospective data about parental socioeconomic position was used. The validity of such retrospective reports may be questionable and may vary according to the age of the respondent. A review of the validity of retrospective responses showed that such reports can be used, although responses concerning adverse conditions are likely to be substantially biased [72,73]. As our measure is not focused on interpretation, such as experiencing financial difficulty, its validity and reliability is likely to be better. Since our key sociodemographic and socioeconomic determinants of sleep were based on current position, these results are less prone to bias. Furthermore, all indicators asked about concrete details of sociodemographic and socioeconomic circumstances that did not involve judgments about personal situation, perceived conditions, or experiences of socioeconomic disadvantage that are more difficult to interpret.

Fourth, since income and employment status, in particular unemployment, had the most consistent associations with sleep duration and insomnia-related symptoms, this raises a question about the mechanisms and role of economic difficulties. Previous studies suggest that economic difficulties exist at all income levels, even among affluent employed populations [74,75], and have adverse effects on sleep [7], and other behaviours [75] after other socioeconomic circumstances are taken into account. Thus, it is possible that the associations found in this study are also explained by greater economic difficulties and related financial and other psychosocial strain.

Fifth, the number of cases with complete data for all sociodemographic and socioeconomic circumstances and sleep varied slightly among our models. However, complete case analyses produced similar results to those reported in our study, suggesting that the estimates are valid. Further control analysis including missing responses as a separate category also produced similar results (data not shown). Thus, we preferred to retain the full sample and use all data available for each analysis without redundant exclusions.

Sixth, ethnicity or race were not assessed as part of the sociodemographic framework as in many other studies [1,2,48]. However, ethnic groups are very small in the population (less than 1%) and the data can be considered very homogeneous with this respect.

Seventh, it might be assumed that the use of hypnotic drugs interferes with the reported associations among sleep duration, insomnia-related symptoms and sociodemographic and socioeconomic circumstances. For example, if hypnotic drug use reduced insomnia-related symptoms and lengthened sleep, and if such medication was disproportionally distributed among socioeconomic groups, this would distort our examination of the associations among sleep and sociodemographic and socioeconomic circumstances. Nonetheless, we conducted control analyses adjusting for hypnotic use (data not shown). The contribution hypnotic drugs made to the associations among sociodemographic and socioeconomic circumstances and sleep was small. Prevalence of hypnotic drug use was 7% and it only partly captures insomnia-related symptoms in these data. Additionally, alcohol may be used as sleep aid, but it also adversely affects sleep maintenance [76]. However, the associations are complex [77-79]. In our sensitivity analyses, adjustment for alcohol had negligible contribution to the examined associations. Further examination of the associations between sleep and alcohol drinking patterns was out of the scope of this study.

Eighth, it should be noted that as these data were collected a decade ago, it is possible that changes occurred in e.g. use of electric media could adversely affect sleep and limit generalizability of the findings to the current situation [80-82]. However, it is of note that our focus was on lifecourse sociodemographic determinants of insomnia-related symptoms and sleep duration among adult Finns. These determinants and patterns of the associations are unlikely to be largely affected by the changes in the use of electronic media. If such media usage is disproportionally distributed across the examined socioeconomic groups, this might suggest that our estimates for e.g. high educated high income participants with potentially more exposure to electronic media are conservative or that the inequalities might be narrowed. Further research is needed to elaborate these issues.

#### Strengths

The strength of this study is that we used a large amount of nationally representative data about the adult population. The results can, therefore, be generalized at the population level in Finland. Generalizability to countries with different socioeconomic structure should be cautious. Moreover, since the data are representative of the general Finnish population, these results provide novel evidence about the distribution of sleep duration among population subgroups and point key groups for insomnia-related symptoms. We also used weighted analyses to improve the generalizability of the findings. A further strength is the availability of a variety of sociodemographic and socioeconomic circumstances over life course. We had data about childhood socioeconomic position and we were therefore able to show that the associations among current socioeconomic circumstances are independent of childhood socioeconomic position. Additionally, we were able to focus on two key characteristics of sleep: quantity and quality.

## Conclusions

Disadvantaged socioeconomic position in adulthood, in particular low income, being unemployed, or being on a disability pension, are associated with poor sleep. When promoting optimal sleep duration and better sleep quality, families with a low income level, disability retirees, and unemployed people should be targeted. Additionally, unmarried adults may be more likely to have poor sleep. In contrast, adult Finns who are married, have children, are employed, have a high income and live in urban areas are most likely to have optimal sleep duration and the best sleep quality. Finally, while poor sleep is connected to ill-health, social factors are important determinants of poor sleep alongside health status. These findings warrant future research to examine the extent to which socioeconomic differences in sleep quantity and quality contribute to persistence and widening of inequalities in health in populations.

## Competing interests

The authors declare to have no competing interests.

## Authors’ contributions

Each author has contributed to the planning of the study and analysis, commented on the manuscript text, as well as approved submission of the final version. LS-J conducted the analyses, TL drafted the first version of the manuscript and helped in the analyses.

## Pre-publication history

The pre-publication history for this paper can be accessed here:

http://www.biomedcentral.com/1471-2458/12/565/prepub
